# Synthesis of a 2''-Deoxy-β-GalCer

**DOI:** 10.3390/molecules190710090

**Published:** 2014-07-10

**Authors:** Meena S. Thakur, Archana Khurana, Mitchell Kronenberg, Amy R. Howell

**Affiliations:** 1 Department of Chemistry, University of Connecticut, 55 N. Eagleville Rd., Storrs, CT 06269, USA; 2 La Jolla Institute of Allergy and Immunology, 9420 Athena Circle, La Jolla, CA 92037, USA

**Keywords:** β-GalCer, antigens, NKT cells, CD1d

## Abstract

Structural studies of ternary complexes of CD1d/glycosyl ceramides/*i*NKT cells and CD1d/sulfatide/sulfatide reactive Type II NKT cells have shown how the polar moieties on the glycolipids interact with both the antigen presenting protein (CD1d) and the T cell receptors. However, these structures alone do not reveal the relative importance of these interactions. This study focuses on the synthesis of the previously unknown 2''-deoxy-β-galactosyl ceramide **2**. This glycolipid is also evaluated for its ability to stimulate *i*NKT cells and sulfatide-reactive Type II NKT cells.

## 1. Introduction

α-Galactosyl ceramide (α-GalCer) and related compounds have garnered increasing attention in the last decade and a half because of their ability to elicit immune responses via CD1d mediated activation of natural killer T (NKT) cells [1,2,3,4,5]. NKT cells are a heterogeneous group of T lymphocytes that express a T cell antigen receptor (TCR) and that perform functions related to both innate and adaptive immunity, including defense against microbes, maintenance of immune tolerance and anti-tumor responses. While most T lymphocytes respond to peptides bound to antigen presenting molecules encoded in the Major Histocompatibility Complex (MHC), a defining characteristic of NKT cells is their recognition of lipid/glycolipid antigens presented by CD1d, which is related to the MHC antigen presenting proteins. CD1d-reactive NKT cells are split into two classes, Type I and Type II. Type I NKT (or *i*NKT) cells are characterized by the expression of an invariant (*i*) TCRα chain paired with a limited number of TCRβ chains, and these cells have been extensively studied [6,7,8,9,10,11]. They recognize various lipid containing antigens, particularly glycosphingolipids. On the other hand, the Type II NKT cells have diverse TCRs and are not as well characterized, although recent attention has focused on the role of sulfatide-reactive cells that belong to this group [12,13,14,15,16,17]. 

**Figure 1:**
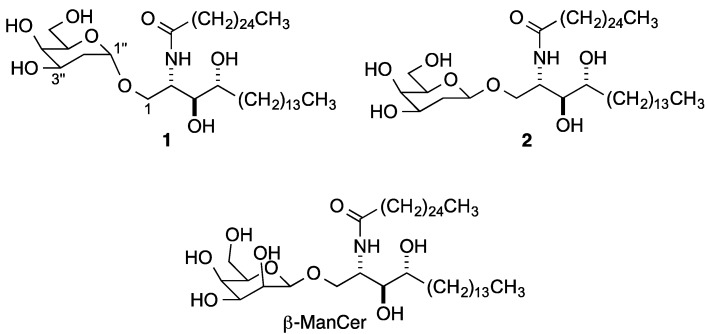
2''-Deoxy galactosyl ceramides **1** and **2** and β-ManCer.

The understanding of the role of the sugar and the ceramide in influencing *i*NKT cell responses has been greatly enhanced by both structure activity relationship (SAR) studies [7,9,10,18,19] and by the availability of an ever increasing number of binary (glycolipid/CD1d) [20,21,22] and ternary (NKT cell TCR/glycolipid/CD1d) crystal structures [23,24,25,26,27,28]. Again, SAR and structural data [29,30,31] for Type II NKT cells lag those for Type I cells. Structural studies show that the lipid moiety of the glycolipids is buried in two pockets of CD1d and that it is predominantly the sugar that interacts with the TCR, which recognizes a composite of the sugar and the surface of the CD1d protein. Although the hydrogen bonding interactions of both CD1d and the NKT cell TCR with the CD1d-exposed sugar moiety are well-characterized, access to deoxysugar analogs has provided a nuanced understanding of the relative influence of each carbohydrate OH and the possibility for fine specificity in interactions [26]. Ternary Type I and Type II structures show that the OH on C2'' has hydrogen bonding interaction with both CD1d and the NKT cell TCR for α-galactosyl ceramides (α-GalCers) and sulfatides (which have β-linked sugars). Indeed, 2''-deoxy-α-GalCer **1** (Figure 1) does not activate Type I NKT cells [32]. Interestingly, however, both β-glucosyl ceramide (β-GluCer) [33] and β-mannosyl ceramide (β-ManCer) [34] have been recently reported to stimulate Type I NKT cells. To our knowledge, no 2''-deoxy-β-glycosyl ceramide has been prepared or evaluated. Herein we report the preparation of 2''-deoxy-β-GalCer **2** as a tool for further developing an understanding of NKT cell activation.

**Figure 2:**
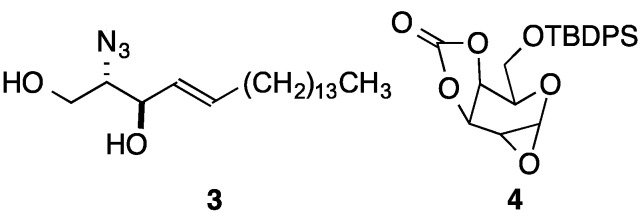
β-Glycosylation partners.

The synthesis of 2''-deoxy-β-glycosides is challenging. If glycosylations targeting such compounds are conducted on already deoxygenated sugar donors, which lack the functionality needed to exploit anchimeric assistance, the stereoselectivity can be low. Moreover, the glycosidic bond is more labile due to the lack of an electron withdrawing C2'' substituent [35]. The most common approach involves the use of a heteroatom substituent at C2, followed by its reductive removal subsequent to glycosylation [36,37,38,39,40,41,42,43,44,45]. Other methods include the use of α-glycosyl phosphites [46,47], S_N_2 reactions of α-glycosyl halides [48,49,50], Pd-catalyzed glycosylation reactions [51], the use of glycosyl imidates under oxidative conditions [52], and anomeric O-alkylation/arylation [53]. Although many of these approaches provided good yields and stereoselectivities with some acceptors, our biggest concern centered on the well-known decreased reactivity of either ceramide or sphingoid base acceptors in comparison to coupling partners used in the majority of these studies. Danishefsky and coworkers had used azidosphingosine **3** in a reaction with 1α,2α-oxirane **4** (Figure 2) derived from galactal, and the glycosylation gave 60% isolated yield of the β-anomer [54]. Although there was not a subsequent deoxygenation in this case, the group has used tin mediated deoxygenations in many other cases. However, our previous experience with difficult purifications in tin-mediated deoxygenations of glycolipids led us to consider an alternative approach.

**Scheme 1:**
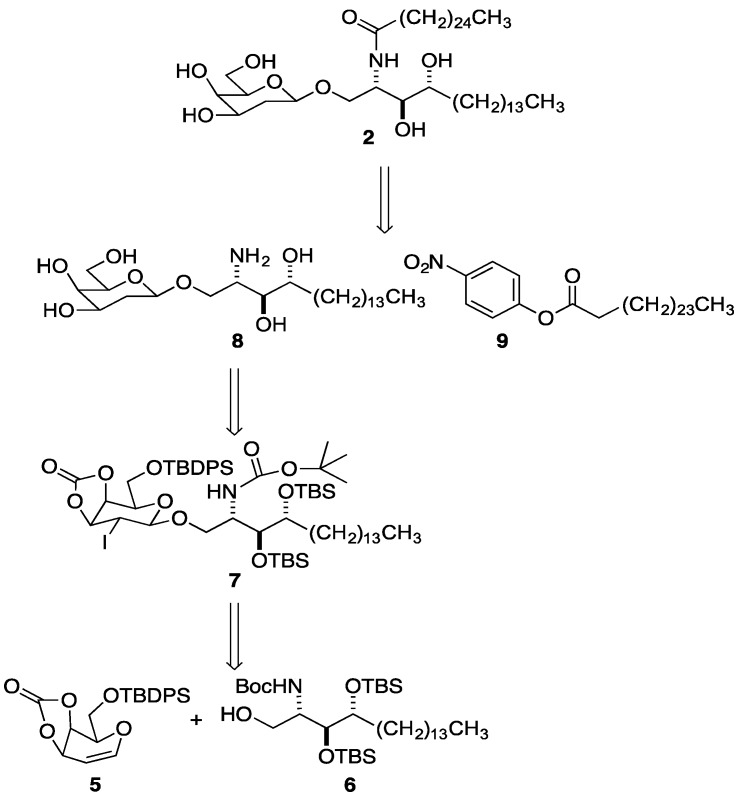
Strategy for the synthesis of 2''-deoxy-β-GalCer 2.

We elected to employ Magnusson’s NIS promoted glycosylation [55] in combination with Danishefsky’s observation that beta-selectivity in 1,2-anhydrosugars was sometimes enhanced with a carbonate (as compared to benzyl groups or an isopropylidene) [56]. Most approaches to glycosyl ceramides involve coupling of a sugar and ceramide or of a sugar and sphingoid base, followed by acylation. The latter is the pathway we chose (Scheme 1). 

Thus, the strategy was to glycosylate via iodoetherification of galactal **5** with phytosphingosine alcohol **6**. Our initial plan was to do a multi-stage deprotection/removal of the iodide of **7** to provide fully deprotected glycosylated phytosphingsine **8**. Final acylation with activated cerotic acid ester **9** would achieve the synthesis of 2''-deoxy-β-GalCer **2**.

## 2. Results and Discussion

Glycosyl donor **5** was prepared in two steps from d-galactal as described by Danishefsky and coworkers [54]. Glycosyl acceptor **6** was synthesized in three steps from phytosphingosine as previously reported [57]. With donor **5** (1 equiv.) and acceptor **6** (2 equiv.) in hand, NIS-induced glycosylation in CH_2_Cl_2_ at room temperature was attempted. The reaction was stirred overnight, and TLC analysis showed consumption of **5**. However, separation of the desired product **7** from the excess of acceptor **6** using column chromatography was extremely difficult, resulting in a poor isolated yield of **7**. A reaction with acceptor **6** as the limiting reactant was sluggish, not going to completion even after 7 days. 

**Scheme 2:**
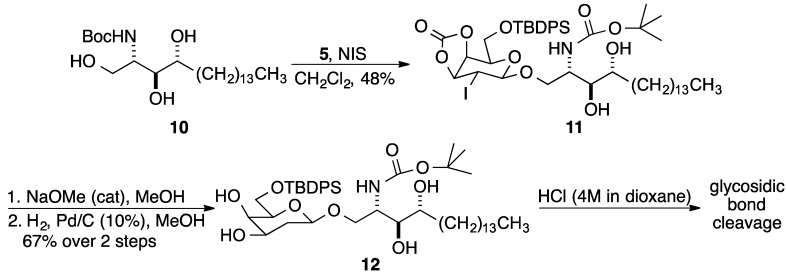
Initial attempt to prepare **2**.

In order to have an acceptor that was more easily separated from the product, NIS-induced glycosylation reaction using acceptor **10** was tried. The reaction was complete in 48 h, and β-glycosylated phytosphingosine **11** was isolated in 48% yield. Compound **11** was then treated with catalytic NaOMe in MeOH, followed by deiodohydrogenation using palladium on carbon (10 wt %) in one pot, to give compound **12** in 67% yield (Scheme 2). However due to the absence of a functionalizable group at C2, cleavage of the anomeric bond was observed in the subsequent Boc deprotection reaction using HCl (4 M in dioxane). Hence the strategy needed to be modified.

**Scheme 3:**
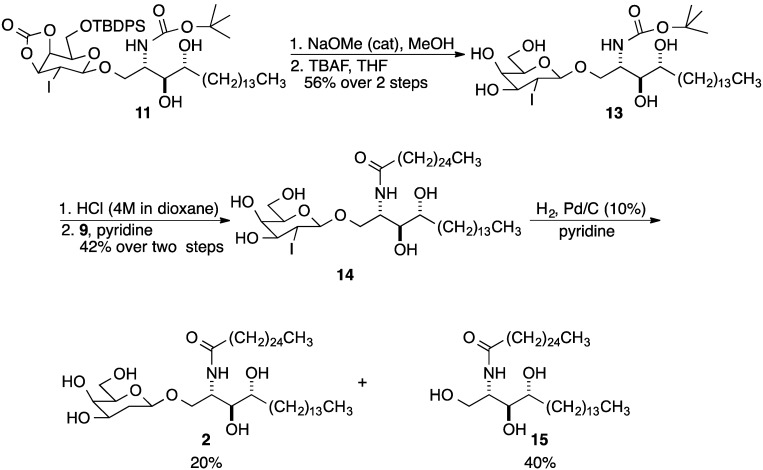
Preparation of **2**.

Glycosylated product **11** was subjected to a one pot, two-stage deprotection (Scheme 3). First, the cyclic carbonate group was removed using cat NaOMe in MeOH; removal of the TBPDS group using TBAF (1.0 M solution in THF), then gave **13** in 56% yield over two steps. Boc cleavage, followed by *N*-acylation using the *para*-nitrophenol ester **9** of cerotic acid in pyridine, afforded **14** in 42% yield. Deiodohydrogenation using palladium on carbon (10 wt %) concluded the synthesis of **2**. A substantial quantity of ceramide **15** was recovered due to the cleavage of the anomeric bond. Nevertheless, there was sufficient material to evaluate 2''-deoxy-β-GalCer **2** for its ability to stimulate both Type I and sulfatide-active Type II NKT cells.

2''-Deoxy-β-GalCer **2** was assessed for its ability to stimulate *i*NKT cells using well-characterized immortalized cells or hybridomas. Hybridoma 1.2 is a well characterized iNKT cell hybridoma [58] that has the common Vα14 chain characteristic of this population, paired with Vβ8.2, the most common TCR β chain expressed by *i*NKT cells. The type II NKT cell hybridomas XV19 (19) and VIII24 (24) were also tested. Hybridoma 19 is reactive to sulfatide, lyso-sulfatide and other antigens [14,59], and it has a TCR containing Vα1 and Vβ16. Hybridoma 24 has a TCR containing Vα3 and Vβ9 and it is CD1d autoreactive for antigens that have not been characterized.

To assay for antigenic potency, a cell-free antigen presentation assay was employed [60]. The assay measures the secretion of interleukin 2 (IL-2) by T cell hybridomas that have been cultured in microwells coated with complexes of CD1d with glycolipids. IL-2 production correlates directly with the strength of TCR stimulation, and because of the high density of lipid-CD1d complexes that can be achieved on the coated plate, this test provides the most sensitive assay for measuring *i*NKT cell responses. 

**Figure 3:**
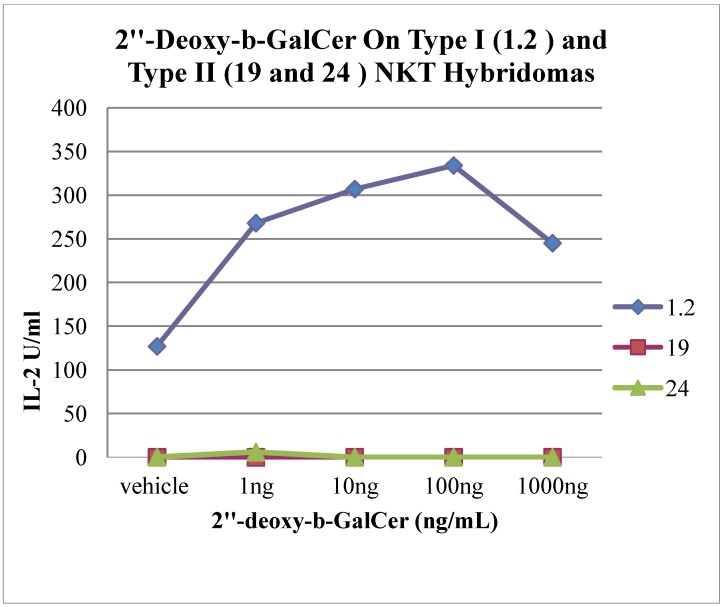
Type I and Type II NKT cell hybridoma responses to 2''-deoxy-β-GalCer **2**.

Figure 3 shows that hybridoma 1.2 weakly reacted to CD1d coated plates loaded with 2''-deoxy-β-GalCer **2**. There is a background reactivity of 127 units/mL of IL-2 released to CD1d molecules obtained from insect cells, which are loaded primarily with phosphatidyl choline [61], but this is more than doubled when the cells were cultured with CD1d molecules loaded with 100 ng/mL of 2''-deoxy-β-GalCer **2**. By contrast, α-GalCer stimulated approximately 10-fold more IL-2 release at a similar concentration. In some assays, however, this 2''-deoxy-β-GalCer **2** reactivity could not be detected, and a weak and somewhat inconsistent stimulation was observed when using antigen presenting cells expressing very high amounts of CD1d or when 1.4, a different *i*NKT cell hybridoma, was tested (data not shown). The pattern was very different with the Type II NKT cell hybridomas (19 and 24), which were completely unreactive to plates coated with CD1d that was not loaded, or loaded with 2''-deoxy-β-GalCer **2**.

## 3. Experimental

### 3.1. General Information

Melting points were determined in open Pyrex capillary tubes and are uncorrected. Specific rotations [α]_D_ were recorded on a JASCO DIP-1000 polarimeter using the sodium D-line as a source, and the concentration (*c*) is expressed in units of g per 100 mL. IR spectra were recorded on a FT-IR spectrometer using KBr. NMR spectra were recorded on a Bruker Avance DRX-400 (400 MHz ^1^H, 100 MHz ^13^C), Bruker Avance (500 MHz ^1^H, 125 MHz ^13^C), or Bruker Avance (300 MHz ^1^H, 75 MHz ^13^C) spectrometer. The ^1^H and ^13^C chemical shifts are reported in parts per million (ppm) from TMS. The following abbreviations are used for peak multiplicities s (singlet); d (doublet); t (triplet); q (quartet); m (multiplet); dd (doublet of doublets); br s (broad singlet). Coupling constants, *J*, are reported in hertz (Hz). Low-resolution mass spectra were obtained on a HP 5970 series GC-MSD system and are reported in units of mass/charge. HRMS were obtained on a JMS-AX505HA spectrometer at the University of Notre Dame. 

*p-Nitrophenyl hexacosanoate* (**9**). A mixture of hexacosanoic acid (1.0 g, 2.5 mmol) and thionyl chloride (12.5 mL) was heated at 90 °C under N_2_ for 12 h. The excess thionyl chloride was distilled off, and traces were removed under high vacuum. The residue was dissolved in dry THF (25 mL), and the solution was added drop-wise to a stirred solution of 4-nitrophenol (0.76 g, 5.5 mmol) and a catalytic amount of DMAP in freshly distilled pyridine (25 mL) under N_2_ at −5 °C. After 1 h at −5 °C the cooling bath was removed, and the mixture was stirred for 12 h at rt. The reaction mixture was then concentrated, and the residue was taken up in CH_2_Cl_2_ and preloaded on silica gel. Purification by flash chromatography on silica gel (petroleum ether/EtOAc 98:2 to 95:5) provided *p*-nitrophenyl hexacosanoate (**9**) as a white powder (0.80 g, 61%): mp 87–88 °C; IR (KBr) 2916, 2849, 1743, 1620, 1593, 1535, 1472, 1348, 1206, 1138 cm^−1^; ^1^H-NMR (CDCl_3_) δ 8.29 (d, *J* = 9.1 Hz, 2H), 7.30 (d, *J* = 9.3 Hz, 2H), 2.62 (t, *J* = 7.5 Hz, 2H), 1.78 (m, 2H), 1.28 (m, 44H), 0.90 (t, *J* = 6.8 Hz, 3H); ^13^C-NMR (CDCl_3_) δ 171.3, 155.5, 145.2, 125.2, 122.4, 34.3, 31.9, 29.7, 29.7, 29.6, 29.6, 29.4, 29.4, 29.4, 29.2, 29.0, 24.7, 22.7, 14.1; MS (EI) *m/z*: 279, 167, 149, 104, 70, 57; Anal. Calcd for C_32_H_55_NO_4_: C, 74.23; H, 10.71; N, 2.71, found: C, 74.22; H, 10.46; N, 2.64.

*(2S,3R,4R)-2-(N-tert-Butoxycarbonyl)amino-1-O-[6-O-(tert-butyldiphenylsilyl)-2-deoxy-2-iodo-3,4-oxacarbonyl-β-d-galactopyranosyl]-1,3,4-octadecantriol* (**11**). 1,5-Anhydro-6-*O*-(*tert*-butyl-di­phenylsilyl)-2-deoxy-d-*lyxo*hex-1-enopyranose-3,4-carbonate (**5**) [54] (0.29 g, 0.71 mmol) and *N*-(*tert*-butoxy­carbonyl) phytosphingosine (0.59 g, 1.4 mmol) (**10**) [62] were added to dry CH_2_Cl_2_ (12 mL) under N_2_ in a foil covered flask. *N*-Iodosuccinimide (0.35 g, 1.6 mmol) was added to the mixture, which was then stirred at rt for 48 h. Then, 10% aq. Na_2_S_2_O_3_ (20 mL) was added, and the mixture was stirred vigorously for 5 min. The mixture was then diluted with EtOAc (100 mL) and transferred to a separatory funnel. The aqueous layer was discarded, and the organic layer was washed with 10% aq. Na_2_S_2_O_3_ (10 mL) and brine (10 mL) and dried (MgSO_4_). The solvents were removed, and the residue was then subjected to gravity column chromatography (petroleum ether/EtOAc 80:20), giving (2*S*,3*R*,4*R*)-2-(*N*-*tert*-butoxycarbonyl)amino-1-*O*-[6-*O*-(*tert*-butyldiphenylsilyl)-2-deoxy-2-iodo-3,4-oxacarbonyl-β-d-galactopyranosyl]-1,3,4-octadecantriol (**11**) as a white solid (0.32 g, 48%): mp 55–57 °C; [α]25D −4.49 (*c* 1.5, CH2Cl2); IR (KBr) 3419 (br), 3072, 2926, 2854, 1818, 1699, 1174, 1114, 702 cm−1; 1H-NMR (CDCl3) δ 7.65 (m, 4H), 7.45 (m, 6H), 5.17 (dd, *J* = 7.4, 5.6 Hz, 1H), 5.14 (m, 1H), 4.80 (dd, *J* = 9.0, 9.0 Hz, 2H), 4.16 (m, 2H), 4.03 (dd, *J* = 5.8, 5.8 Hz, 1H), 3.91 (m, 2H), 3.82 (br s, 1H), 3.59 (m, 3H), 2.85 (br s, 1H), 2.14 (br s, 1H), 1.65 (m, 1H) 1.43 (s, 10H), 1.26 (s, 24H), 1.08 (s, 9H), 0.89 (t, *J* = 6.7 Hz, 3H); 13C-NMR (CDCl3) 156.2, 153.4, 135.5, 135.5, 132.8, 132.5 130.1, 128.1, 101.5, 80.1, 79.7, 74.6, 73.4, 73.2, 71.8, 70.0, 62.2, 51.5, 32.5, 32.1, 29.8, 29.5, 28.6, 27.0, 26.1, 22.9, 19.4, 14.3; HRMS (ESI) *m/z* calcd for C46H73INO10Si [M+H]^+^ 954.4043, found 954.4051.

*(2S,3R,4R)-2-(N-tert-Butoxycarbonyl)amino-1-O-[6-O-(tert-butyldiphenylsilyl)-2-deoxy-β-d-galacto­pyranosyl]-1,3,4-octadecantriol* (**12**). (2*S*,3*R*,4*R*)-2-(*N*-*tert*-Butoxycarbonyl)amino-1-*O*-[6-*O*-(*tert*-butyldiphenylsilyl)-2-deoxy-2-iodo-3,4-oxacarbonyl-β-d-galactopyranosyl]-1,3,4-octadecan-triol (**11**) (0.21 g, 0.22 mmol) was dissolved in MeOH (1.1 mL) at rt under N2, followed by the addition of a catalytic amount of NaOMe (0.5 M in MeOH) (0.030 mL, 0.013 mmol). The reaction mixture was stirred at rt for 0.5 h. The reaction mixture was concentrated, and the residue was dissolved in MeOH/EtOAc (5:1, 6.5 mL), followed by the addition of Et3N (0.04 mL) and Pd/C (10%, 86 mg). The mixture was stirred vigorously overnight under H2 (1 atm) and then filtered through celite and concentrated. The crude reaction mixture was purified by gravity column chromatography (CH2Cl2/MeOH 98:2), giving (2*S*,3*R*,4*R*)-2-(*N*-*tert*-Butoxycarbonyl)amino-1-*O*-[6-*O*-(*tert*-butyldiphenylsilyl)-2-deoxy-2-iodo-3,4-oxacarbonyl-β-d-galactopyranosyl]-1,3,4-octadecantriol (**12**) as a clear oil (0.12 g, 67%): IR (KBr) 3419 (br), 2926, 1699, 1174, 1114 cm−1; 1H-NMR (CDCl3) δ 7.63 (m, 4H), 7.37 (m, 6H), 5.20 (d, *J* = 8.6 Hz, 1H), 4.33 (d, *J* = 9.2 Hz, 1H), 4.14 (d, *J* = 8.4 Hz, 1H), 3.82 (m, 4H), 3.57 (m, 5H), 3.35 (dd, *J* = 5.7, 5.7 Hz, 1H), 3.13 (dd, *J* = 14.6, 7.3 Hz, 1H), 3.07 (m, 1H), 2.60 (br s, 1H), 1.95 (m, 1H), 1.65 (m, 2H), 1.42 (m, 3H), 1.39 (s, 9H), 1.22 (br s, 22H), 1.01 (s, 9H), 0.84 (t, *J* = 6.6 Hz, 3H); 13C-NMR (CDCl3) δ 155.9, 135.8, 135.7, 133.2, 133.2, 130.1, 128.1, 100.7, 79.9, 75.2, 74.7, 73.0, 69.1, 68.7, 67.6, 63.3, 53.5, 51.4, 46.5, 35.2, 32.3, 32.1, 29.9, 29.6, 28.6, 27.0, 26.2, 22.9, 19.4, 14.3, 8.8; HRMS (ESI) *m/z* calcd for C45H76NO9Si [M+H]^+^ 802.5284, found 802.5300.

*(2S,3R,4R)-2-(N-tert-Butoxycarbonyl)amino-1-O-[6-O-(tert-butyldiphenylsilyl)-2-deoxy-β-d-galacto­pyranosyl]-1,3,4-octadecantriol* (**13**). (2*S*,3*R*,4*R*)-2-(*N*-*tert*-Butoxycarbonyl)amino-1-*O*-[6-*O*-(*tert*-butyldiphenylsilyl)-2-deoxy-2-iodo-3,4-oxacarbonyl-β-d-galactopyranosyl]-1,3,4-octadecantriol (**11**) (0.10 g, 0.1 mmol) was dissolved in MeOH (0.5 mL) at rt under N2, followed by the addition of a catalytic amount of NaOMe (0.5 M in MeOH) (0.01 mL, 0.006 mmol). The reaction mixture was stirred at rt for 0.5 h. The reaction mixture was concentrated, and the residue was dissolved in dry THF (0.5 mL). The reaction mixture was cooled to 0 °C under N2, and TBAF (1.0 M, THF) (0.2 mL, 0.2 mmol) was added dropwise. The cooling bath was removed, and the reaction mixture was allowed to stir at rt for 1 h. The mixture was then concentrated and purified by gravity column chromatography (CH2Cl2/MeOH 95:5) giving (2*S*,3*R*,4*R*)-2-(*N*-*tert*-Butoxycarbonyl)amino-1-*O*-[2-deoxy-2-iodo-β-d-galactopyranosyl]-1,3,4-octa-decantriol (**13**) as a white solid (0.040 g, 56%): mp 140–142 °C; [α]^25^D −26.1 (*c* 0.64, CH2Cl2/MeOH, 90:10); IR (KBr) 3430, 2924, 2854, 1716, 1698, 1685 cm-1; 1H-NMR (CDCl3) δ 5.49 (d, *J* = 9.7 Hz, 1H), 4.61 (m, 2H), 4.40 (m, 2H), 4.26 (m, 1H), 4.19 (br s, 1H), 4.09 (dd, *J* = 9.7, 9.7 Hz, 1H), 3.93–3.74 (m, 8H), 3.64 (br s, 1H), 3.54 (m, 1H), 2.57 (br s, 1H), 1.54 (m, 2H), 1.46 (br s, 10H), 1.28 (s, 22H), 0.90 (t, *J* = 6.0 Hz, 3H); 13C-NMR (CDCl3) 156.0, 104.1, 80.1, 77.4, 75.5, 75.4, 74.0, 73.1, 69.7, 61.9, 51.2, 35.5, 32.2, 30.0, 30.0, 29.9, 29.6, 28.7, 26.5, 22.9, 14.3; HRMS (ESI) *m/z* calcd for C29H56INNaO9 [M+Na]^+^ 712.2892, found 712.2914. 

*(2S,3R,4R)-2-(N-tert-Butoxycarbonyl)amino-1-O-[6-O-(tert-butyldiphenylsilyl)-2-deoxy-β-d-galacto­pyranosyl]-1,3,4-octadecantriol* (**14**). (2*S*,3*R*,4*R*)-2-(*N*-*tert*-Butoxycarbonyl)amino-1-*O*-[2-deoxy-2-iodo-β-d-galactopyranosyl]-1,3,4-octa-decantriol (**13**) (0.037 g, 0.054 mmol) was dissolved in dry 1,4-dioxane (0.5 mL) at rt under N2. HCl (4 M, dioxane) (0.5 mL) was added, and the reaction mixture was stirred at rt for 40 min. The reaction mixture was then brought to pH 8 by adding saturated aqueous NaHCO3 and was stirred for a further 30 min. A solid appeared and was subsequently filtered, dissolved in pyridine, then concentrated. The crude solid was dissolved in a minimum quantity of dry pyridine (~1.0 mL) at rt under N2. The *p*-nitrophenol ester (**9**) of cerotic acid (0.026 g, 0.050 mmol), dissolved in a minimum quantity of pyridine (~0.5 mL), was added to the reaction mixture, and the solution was stirred for 20 h at rt under N2. The reaction mixture was concentrated, and the residue was purified by gravity column chromatography (CH2Cl2/MeOH 93:7) giving (2*S*,3*R*,4*R*)-1-*O*-[2-deoxy-2-iodo-β-d-galactopyranosyl]-1,3,4-octadecantriol (**14**) as a white solid (0.20 g, 42%): mp 120 °C; [α]25D 80.2 (*c* 0.05, pyridine); IR (KBr) 3430, 2957, 2851, 1651, 1114, 1067 cm−1; 1H-NMR (pyridine-d5) δ 8.22 (d, *J* = 8.1 Hz, 1H), 6.05 (br s, 5H), 5.12 (m, 2H), 4.82 (m, 2H), 4.50−4.35 (m, 5H), 4.23 (m, 2H), 4.09 (dd, *J* = 6.9, 6.9 Hz, 1H), 2.50 (m, 2H), 2.24 (m, 1H), 1.95−1.85 (m, 4H), 1.71 (m, 1H), 1.30 (m, 66H), 0.87 (m, 6H); 13C-NMR (pyridine-d5) 173.0, 104.4, 77.1, 73.3, 75.1, 72.8, 70.5, 61.6, 51.9, 39.7, 36.7, 33.0, 31.8, 30.1, 29.9, 29.6, 29.3, 26.4, 26.1, 22.7, 14.0; HRMS (ESI) *m/z* calcd for C50H99INO8 [M+H]^+^ 968.6410, found 968.6434. 

*(2S,3R,4R)-2-(N-tert-Butoxycarbonyl)amino-1-O-[6-O-(tert-butyldiphenylsilyl)-2-deoxy-β-d-galacto­pyranosyl]-1,3,4-octadecantriol* (**2**). Pd/C (10%, 29 mg) was added to a stirred solution of (2*S*,3*R*,4*R*)-1-*O*-[2-deoxy-2-iodo-β-d-galactopyran­osyl]-2-hexacosanoylamino-1,3,4-octadecantriol (**14**) (0.05 g, 0.05 mmol) in pyridine (1.5 mL). The mixture was stirred vigorously overnight under H2 (1 atm) and then filtered through celite and concentrated. The crude reaction mixture was purified by gravity column chromatography (CH2Cl2/MeOH, 93:7), giving (2*S*,3*R*,4*R*)-1-*O*-[2-deoxy-β-d-galactopyranosyl]-2-hexacosanoylamino-1,3,4-octadecane-triol (**2**) as a white solid (9.0 mg, 20%): mp 175 °C; [α]25D −0.74 (*c* 0.37, MeOH/CH2Cl2, 20:80); IR (KBr) 3423, 2919, 2851, 2361, 2337, 1652, cm−1; 1H-NMR (CDCl3:MeOH; 95:5) δ 4.41 (d, *J* = 9.4 Hz, 1H), 4.16 (m, 1H), 3.97 (dd, *J* = 10.8, 4.0 Hz, 1H), 3.82 (dd, *J* = 11.6, 5.8 Hz, 1H), 3.71 (m, 3H), 3.61 (m, 1H), 3.51 (m, 2H), 3.33 (m, 2H), 3.06 (dd, *J* = 5.20, 5.20 Hz, 1H), 1,89 (m, 1H), 1.78 (m, 1H), 1.68–1.36 (m, 6H), 1.18 (s, 66H), 0.80 (t, *J* = 6.60 Hz, 6H); 13C-NMR (CDCl3:MeOH; 95:5) δ 174.2, 100.9, 75.3, 75.0, 72.9, 68.2, 68.6, 62.9, 50.7, 36.8, 34.8, 33.1, 32.1, 26.1, 25.9, 22.8, 22.7, 22.4, 14.1; HRMS (ESI) *m/z* calcd for C50H99NNaO8 [M+Na]^+^ 864.7263, found 864.7250.

### 3.2. Biological Assays

Stimulation of NKT cell hybridomas on microwell plates coated with soluble mouse CD1d was carried out according to published protocols [60,63,64]. Briefly, the amounts of compound indicated in the figure were incubated for 24 h in microwells that had been coated with 1.0 µg of mCD1d. After washing, 5 × 10^4^ to 1 × 10^5^ NKT hybridoma cells were cultured on the plate for 20 h, and IL-2 in the supernatant was measured by enzyme-linked immunoassay (ELISA).

## 4. Conclusions

The synthesis of 2''-deoxy-β-GalCer **2** and evaluation of its ability to stimulate two types of NKT cells have been described. Type II NKT cell hybridoma 19 reacts to sulfatide, as well as β-GalCer [14], and although *i*NKT cells prefer glycosphingolipids with α-linked sugars, it has been recently reported that they react to β-GluCer and β-ManCer. Therefore, it was conceivable that the hybridomas tested would have reactivity for this novel glycosphingolipid. However, only a marginal stimulation for *i*NKT cells was observed. For the sulfatide-reactive Type II NKT cells previously described structural data suggest that the 2''-OH group on the sugar plays a critical role through its interactions with CD1d and the NKT cell receptor. We have confirmed the crucial importance of the 2''-OH for sulfatide-reactive Type II NKT cell stimulation, as 2''-deoxy-β-GalCer did not stimulate the Type II NKT cell hybridomas.
